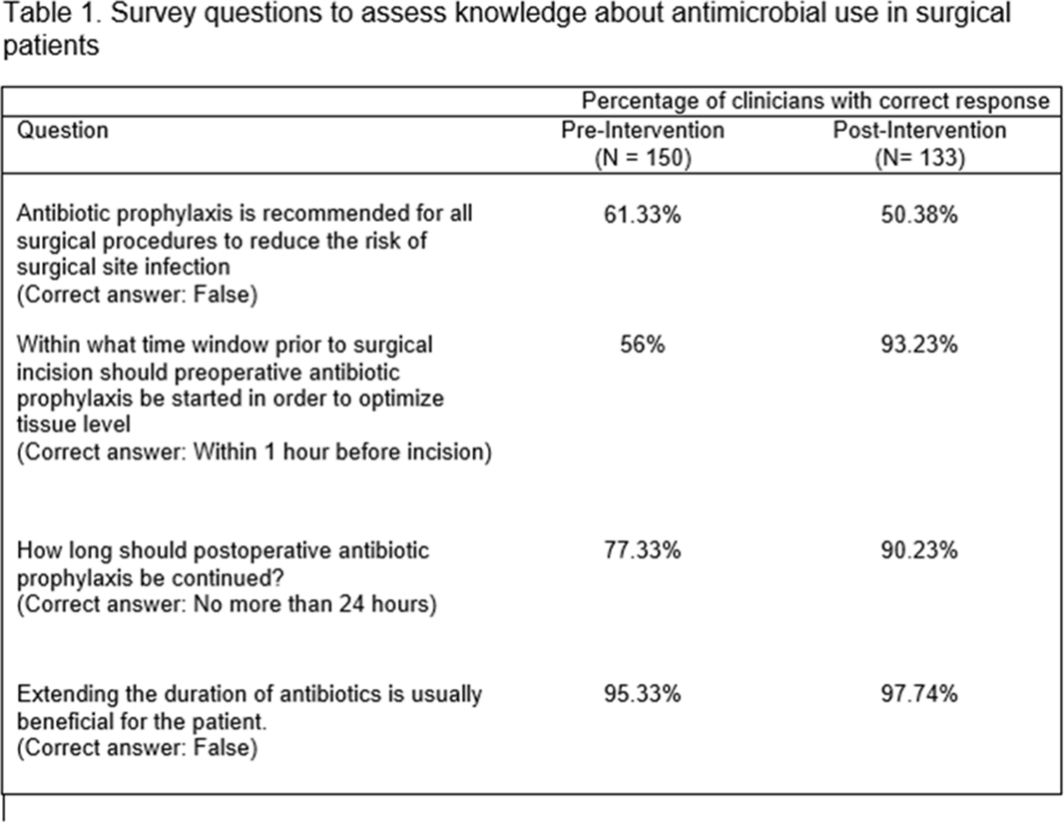# Promoting Antimicrobial Stewardship Education Among Pediatricians Through a Maintenance of Certification Part 4 Quality Impro

**DOI:** 10.1017/ash.2021.53

**Published:** 2021-07-29

**Authors:** Prachi Singh, Brian Lee, Jenna Holmen

## Abstract

**Background:** The rise of antimicrobial resistance has made it critical for clinicians to understand antimicrobial stewardship principles. We sought to determine whether the opportunity to participate in an American Board of Pediatrics Maintenance of Certification Part 4 (MOC4) quality improvement (QI) project would engage pediatricians and improve their knowledge about antimicrobial stewardship. **Methods:** In August 2019, a new clinical algorithm for acute appendicitis, spearheaded by the antimicrobial stewardship program (ASP), was implemented at UCSF Benioff Children’s Hospital Oakland to standardize care and optimize antimicrobial use. Medical staff were invited to participate in a QI project evaluating the impact of this algorithm. Data were collected for the 2 quarters preceding implementation (baseline), for the quarter of implementation (transition period), and for the quarter after implementation. Participants were offered MOC4 credit for reviewing these 3 cycles of data and associated materials highlighting information about antimicrobial stewardship. An initial survey was given to participants to assess their baseline knowledge via 4 questions about antimicrobial use in surgical patients (Table 1). At the conclusion of the QI project, another survey was conducted to reassess participant knowledge and to evaluate overall satisfaction with the project. **Results:** In total, 150 clinicians completed the initial survey. Of these, 44% were general pediatricians and 56% were pediatric subspecialists. Based on years out of training, their levels of experience varied: >20 years in 24%, 11–20 years in 32.7%, 0–10 years in 34.7%, and currently in training in 8.7%. Of the 150 initial participants, 133 (89%) completed the QI project and the second survey. Between surveys, there was significant improvement in knowledge about the appropriate timing and duration of surgical antibiotic prophylaxis (Table 1). Moreover, 88% of participants responded that the QI project was extremely effective in helping them learn about antimicrobial stewardship principles and about ASP interventions. **Conclusions:** Participation in this MOC4 QI project resulted in significant improvement in knowledge about antimicrobial use in surgical patients, and the activity was perceived as a highly effective way to learn about antimicrobial stewardship. QI projects that leverage MOC4 credit can be a powerful tool for engaging pediatricians and disseminating education about antimicrobial stewardship.

**Funding:** No

**Disclosures:** None

**Table 1.**